# Evaluation of the cytotoxic effect of titanium dioxide nanoparticles in human embryonic lung cells

**DOI:** 10.55730/1300-0144.5733

**Published:** 2023-10-12

**Authors:** Olkan AFŞAR, Çağatay OLTULU

**Affiliations:** 1 Department of Pharmaceutical Nanotechnology, Institute of Health Sciences, Trakya University, Edirne, Turkiye; 2 Department of Pharmaceutical Toxicology, Faculty of Pharmacy, Trakya University, Edirne, Turkiye

**Keywords:** Titanium dioxide nanoparticles, oxidative DNA damage, cytotoxicity

## Abstract

**Background/aim:**

Titanium dioxide nanoparticles are widely used in a variety of products, including sunscreens, paints, and ceramics. However, their increasing use has raised concerns about their potential health risks. Titanium dioxide nanoparticles have been shown to have the ability to enter the bloodstream and accumulate in various tissues, reaching the fetus via the placenta. The aim of this study was to investigate the cytotoxic effects of titanium dioxide nanoparticles on a human embryonic lung cell line (HEL 299/An1) and the formation of oxidative DNA damage.

**Materials and methods:**

The cytotoxic effects of brookite-based titanium dioxide nanoparticles (<100 nm) were assessed using the 3-(4,5-dimethyldiazol-2-yl)-2,5 diphenyl tetrazolium bromide (MTT) assay for 24 and 48 h. Cell titanium levels were determined using inductively coupled plasma mass spectrometry. Oxidative DNA damage was assessed by measuring the levels of 8-hydroxy-2-deoxyguanosine (8-OHdG) as a biomarker.

**Results:**

Titanium dioxide nanoparticles caused dose-dependent cytotoxicity in HEL 299/An1 cells. The IC_50_ values were 25.93 μM and 0.054 μM after 24 h and 48 h of exposure, respectively. Cell titanium levels were found to be 25,967 ppb after 24 h and 210,353 ppb after 48 h (p < 0.01). 8-OHdG was detected at 32.96 ng/mL after 24 h of exposure and 17.89 ng/mL after 48 h of exposure.

**Conclusion:**

In our study, it was shown that titanium nanoparticles caused dose-dependent cytotoxicity and oxidative DNA damage in human embryonic lung cells. The nanoparticles also accumulated in cells and were taken up in higher amounts after 48 h of exposure. These findings suggest that titanium dioxide nanoparticles may pose a health risk, especially for pregnant women who may not be aware of their pregnancy. Therefore, it is important to take preventive measures to reduce exposure to these nanoparticles.

## 1. Introduction

Titanium dioxide nanoparticles (TiO_2_ NPs) have entered our lives with the development of nanotechnology. As the production of TiO_2_ NPs increases, the amount entering the environment also increases and concerns about health effects from the increased use of TiO_2_ NP products are growing. TiO_2_ is a polymorphic metal oxide found in nature and has three main crystal phases: brookite, rutile, and anatase. Brookite is the least common crystal phase [1]. TiO_2_ is used in food, cosmetics, toothpaste, implants, medicine, paint, ceramics, and plastics. Although the use of E171 TiO_2_ as a food additive has been prohibited by the European Commission since 2022, it is still used in some countries. Exposure limits for TiO_2_ NPs vary among countries and there is a possibility that these limits will be updated with lower values in the future. Exposure to TiO_2_ NPs can occur during both production and consumption of products. Potential routes of exposure include inhalation and dermal contact in the workplace, as well as oral exposure through consumption. TiO_2_ is absorbed into the body via food and other products, with some absorbed and entering the systemic circulation and the rest excreted [2]. Exposure to TiO_2_ NPs via inhalation was found to have negative effects on the fetus and placenta in a study conducted on pregnant rats [3]. In 2006, the International Agency for Research on Cancer categorized exposure to TiO_2_ through inhalation as a potential human carcinogen (Category 2B). TiO_2_ is used in sun-protective formulations due to its ability to block ultraviolet rays. The stratum corneum in the intact skin prevents the penetration of inorganic substances. It has been reported that TiO_2_ NPs cannot enter through undamaged skin [4]. However, superficial skin damage, including sunburn, can alter skin permeability.

A recent study found that 35-nm coated and uncoated as well as coated 100-nm and 250-nm TiO_2_ NPs could not enter the stratum corneum of intact skin. However, these NPs were observed to penetrate damaged skin [5]. In other studies, it was reported that TiO_2_ NPs penetrated through pores and hair follicles when applied to hairy skin using an oil emulsion in water [6]. The photocatalytic property of TiO_2_ NPs induced by ultraviolet light can be used to degrade pollutants in water and air [7]. For example, TiO_2_ NPs can be used to clean cyanide in wastewater in gold mining [8].

In recent years, TiO_2_ NPs have attracted significant interest due to their unique properties, including excellent photocatalytic activity, UV protection, antibacterial properties, high refractive index, stability and inertness, and biocompatibility. Titanium dioxide nanoparticles have shown promising potential in biomedical applications, including photodynamic therapy, drug delivery, antivirals, biosensors, tissue antibacterials, and dental implants [9]. TiO_2_ can generate reactive oxygen species (ROS) when exposed to ultraviolet light in an aqueous environment [9]. The production of ROS, which can lead to cell death, makes TiO_2_ a promising candidate for photodynamic therapy for the treatment of cancer [9]. TiO_2_ NPs can be used to deliver drugs to specific cells or tissues [10]. This can be done by coating the NPs with targeting molecules that bind to specific receptors on cells. Once the NPs bind to the cells, they can release the drugs, which can then kill the cells or treat a disease. TiO_2_ NPs can also be used to deliver genes to cells. This can be done by coating the NPs with a gene carrier that protects the gene from being damaged. Once the NPs enter the cells, they can release the gene, which can then be expressed by the cells. Gene therapy has the potential to treat a variety of genetic diseases, including cancer, cystic fibrosis, and sickle cell anemia. TiO_2_ NPs can be used to develop biosensors, which are devices that can detect the presence of specific molecules [10]. Biosensors can be used to monitor disease progression, diagnose diseases, and track the effectiveness of treatments [11]. TiO_2_ NPs can be used to develop tissue engineering scaffolds, which are three-dimensional structures that can be used to grow new tissues [12]. Tissue engineering scaffolds can be used to treat a variety of injuries and diseases, including burns, bone fractures, and heart disease. TiO_2_ NPs can be used to improve the biocompatibility of dental implants [10]. Dental implants are devices that are surgically placed into the bone to replace missing teeth. TiO_2_ NPs can be coated onto the surface of dental implants to make them less likely to be rejected by the body. However, there are some concerns about the safety of TiO_2_ NPs. For example, TiO_2_ NPs may be able to enter the bloodstream and travel to other parts of the body, where they could potentially cause damage. More research is needed to fully understand the safety profile of TiO_2_ NPs before they can be widely used in biomedical applications.

Nanoparticles with small sizes (<100 nm) exhibit higher surface area and reactivity, which can increase the probability of migration to secondary organs [13]. Decreasing the size of the nanoparticle increases the toxicity, and crystal morphology is a factor in toxicity. In a study conducted on Chinese hamster ovary cells, it was found that the toxic effects and the occurrence of 8-hydroxy-2-deoxyguanosine (8-OHdG) induced by ultraviolet light were higher for anatase TiO_2_ NPs of 10–20 nm in size compared to 50–60 nm, while for rutile TiO_2_ NPs of 50–60 nm in size, the effects were lower [14]. In another study, it was shown that the lung effects produced by titanium particles administered by intratracheal instillation were linked to the particle surface area [15]. It has been shown that the administration of TiO_2_ NPs intratracheally to rats caused structural and functional disturbances in alveolar macrophages, leading to immunotoxicity [16]. The effect of nanoparticle shape on toxicity was evaluated when TiO_2_ NPs in the form of bipyramids, rods, and platelets were tested on human bronchial epithelial cells using the WST-1 assay, showing that shape affects the toxicity and increases oxidative DNA damage [17]. Several forms of TiO_2_ NPs, including brookite, amorphous, rutile, and anatase, coated with bovine serum albumin and polyethylene glycol were found to reduce viability and proliferation in the MKN-45 human gastric cancer cell line in a time- and dose-dependent manner and trigger apoptosis. Only the brookite TiO_2_ NP group coated with bovine serum albumin reduced invasion [18]. In a study conducted on A549 human lung epithelial cells, it was reported that TiO_2_ nanoparticles of nanorod shape were more cytotoxic than spherical ones of the same surface area and size [19].

Exposure of adult rats to TiO_2_ NPs through inhalation causes the occurrence of ROS and irreversible DNA damage within 7 days, as demonstrated in a previous study [20]. This damage to the DNA leads to modifications in sugars and bases, breaks in single and double helixes, and covalent cross-linking, resulting in the production of DNA damage products. One of the characteristics of guanine among DNA bases is its low oxidation potential, which makes it more vulnerable to oxidative damage. 8-Hydroxyguanine (8-OxoG) and 8-OHdG are formed by the addition of a single oxygen or hydroxyl radical to the guanine base [17]. 8-OHdG is recognized by the base excision repair mechanism and expelled from the DNA chain to the outside of the cell [21]. 8-OHdG is used as an important biomarker to help measure oxidative stress associated with different conditions such as chronic diseases, induction of cancer by radicals, lifestyle, diet, and aging [21].

TiO_2_ NPs have been shown to have the ability to penetrate the bloodstream and accumulate in various tissues via oral, inhalation, and dermal exposure routes [22]. Yamashita et al. conducted a study that showed detection of TiO_2_ NPs in the placenta, liver, and brain of the fetus, which resulted in fetal resorption and developmental retardation when 35-nm rutile TiO_2_ NPs were intravenously applied to mice on the 16th and 17th days of pregnancy [23]. Research has demonstrated that when mice are exposed to TiO_2_ nanoparticles, it results in elevated levels of titanium in the placenta, fetus, and maternal serum. This increase in titanium is associated with a decrease in calcium and zinc levels, ultimately leading to fetal developmental retardation, weight loss, lung development disorders, and a decrease in the number of viable fetuses [24,25]. Exposure of mothers to TiO_2_ NPs can cause accumulation in the placenta and negatively affect its function, and it can reach the fetus, which is sensitive to toxic agents due to its insufficient detoxification capacity and not yet fully developed defense mechanisms [26]. Our study aims to assess the impact of TiO_2_ NPs on DNA damage in HEL 299/An1 cells.

## 2. Materials and methods

### 2.1. Cells and determination of IC_50_ dose using 3-(4,5-dimethyldiazol-2-yl)-2,5 diphenyl tetrazolium bromide (MTT) test

The HEL 299/An1 human embryonic pulmonary cell line was obtained from the Ankara Şap Institute. In the MTT test, a colorimetric method based on the principle of the conversion of yellow-colored MTT tetrazolium salt into insoluble purple crystals by the mitochondria of live cells is utilized, with the color change being determined by spectrophotometry.

A total of 1 × 10^5^ cells were cultured in 96-well plates and maintained at 37 °C, 95% humidity, and 5% CO_2_ for a duration of 24 h [27]. Subsequently, 20 μL of brookite TiO_2_ NPs (Sigma 791326, size: <100 nm; Sigma, Burlington, MA, USA) were applied to separate plates in a dose range of 0.90–400 μM for 24 and 48 h of exposure. All wells were treated with MTT solution of 5 mg/mL (20 μL/well) and incubated for 2 h. Next, the plates were inverted and the medium was aspirated. The absorbance of tetrazolium salt dissolved in 200 μL of DMSO was determined at 492 nm by spectrophotometer (Multiskan GO, Thermo Fischer Scientific, Vantaa, Finland). The obtained data were evaluated with probit analysis using IBM SPSS Statistics 22 (IBM Corp., Armonk, NY, USA) and IC_50_ values were calculated. Cell viability was determined with the following formula: % Cell viability = Sample absorbance value/Control absorbance value × 100.

### 2.2. Determination of titanium quantity by inductively coupled plasma-mass spectrometry (ICP-MS)

**Figure 1:**
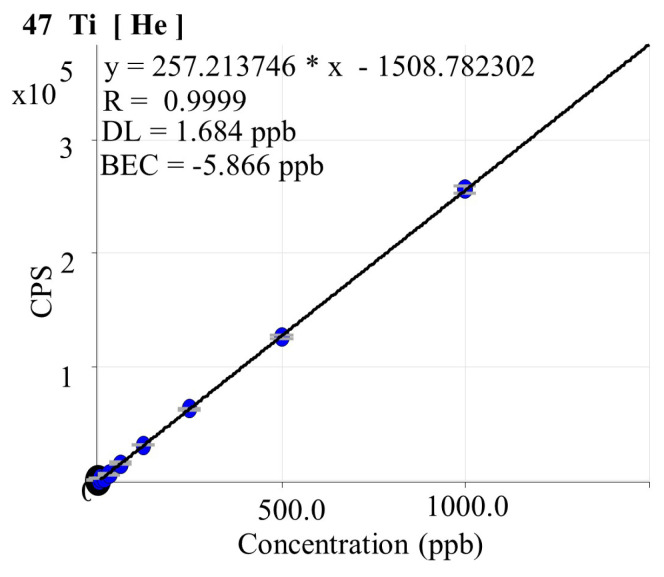
Titanium ICP-MS calibration curve.

Six-well plates were inoculated with 3 × 10^6^ cells, and after exposure to the IC_50_ values for 24 h (25.93 μM) and 48 h (0.054 μM), ICP-MS analysis was conducted [28]. After removing the medium from the cell culture, 1 mL of 65% nitric acid was applied to the cells remaining at the bottom. It was allowed to digest for 24 h and then 9 mL of ultrapure water was added and the solution was read with an Agilent 7700 model ICP mass spectrometer (Agilent Technologies, Waldbronn, Germany). The isotope ^49^Ti was used for titanium detection and ^115^In as the internal standard (Figure 1).

### 2.3. DNA isolation and hydrolysis

Cells were cultured in 6-well plate (3 × 10^6^ cells per well) and exposed to TiO_2_ NPs at the IC_50_ dose for 24 (25.93 μM) and 48 (0.054 μM) h, followed by isolation of DNA using the Pure Link Genomic DNA Mini Kit (Invitrogen, Waltham, MA, USA) referring to the kit’s manual. The purity and quantity of DNA were measured by spectrophotometry and normalized (Nanodrop, Thermo Fisher, Karlsruhe, Germany). The DNA hydrolysis method employed by Crow et al. was utilized, which involved the addition of DNA deferoxamine mesylate for DNA hydrolysis [29]. Nuclease P1 (10 μL, Sigma) was dissolved in 0.3 M sodium acetate at a concentration of 1 U/μL and 1 mM ZnSO_4_ (pH 5.3) was added. The resulting solution was added to DNA dissolved in deferoxamine mesylate [29]. The mixture solution containing undissolved DNA was subjected to vortexing and incubated at 37 °C for 2 h. After another vortexing, it was incubated for an additional 1 h, and then 10 μL of 0.5 M Tris, 0.2 μL of alkaline phosphatase, and 1 mM Na_2_EDTA mixture at pH 8 were added after 20 U/μL and incubated for 1 h [29]. After vortexing, the mixture was incubated at 37 °C for 30 min and vortexed again. Subsequently, the supernatant was obtained for analysis by centrifuging the mixture at 5000 × *g* for 5 min. The DNA quantities were normalized based on the determination of DNA amount and purity (260/280 ratio) before being included in the analysis.

### 2.4. Determination of 8-OHdG content by liquid chromatography-mass spectrometry-mass spectrometry (LC-MS/MS)

After isolating and hydrolyzing the DNA from cells exposed to IC_50_ values of 25.93 μM for 24 h and 0.054 μM for 48 h, we utilized the LC-MS/MS method described by Crow et al. to determine the levels of 8-OHdG [29]. The internal standard used for 8-OHdG detection in LC-MS/MS analysis was H5653 (Sigma). The Agilent 1260 HPLC system (Agilent Technologies) and 6460 Triple Quadrupole mass analyzer (Agilent Technologies) were utilized for analysis. A Poroshell EC C18.6 × 150 mm, 2.7 m column (Agilent Technologies) was attached to the device and set to 40 °C, and 20 μL of sample was run on the device. Two distinct mobile phases were employed. The first mobile phase consisted of ultrapure water with 0.1% formic acid, whereas the second mobile phase was methanol-based. The mobile phase mixture was then sent to the system (0.5 mL/min). Analytes were collected from the column using an elution technique with a methanol gradient.

**Figure 2:**
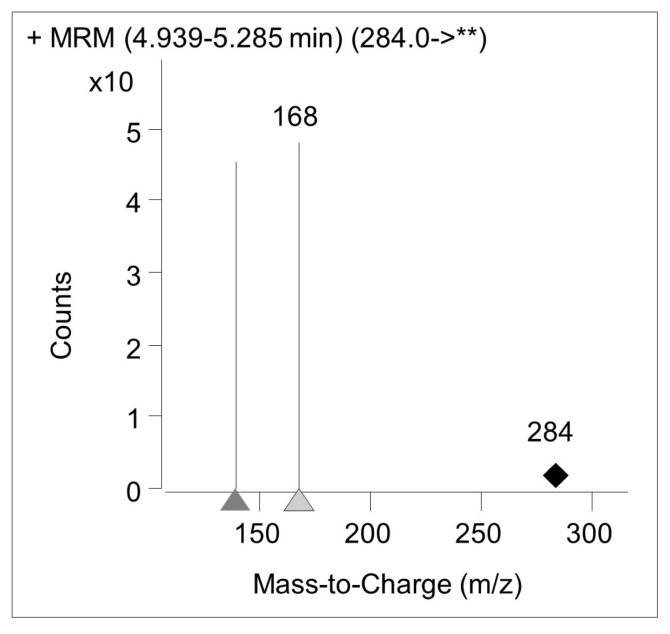
Mass fingerprint of 8-OHdG obtained from LC-MS/MS analysis.

After observing 8-OHdG peaks in the range of 4–5 min, a gradient of 80% methanol solution from line B was applied between 5 and 6 min, a gradient of 30% methanol was applied between 6 and 7 min, and analysis was completed for each sample at 7 min (Figure 2). After the analysis was completed, the column was washed with a 50% methanol solution.

## 3. Results

### 3.1. MTT assay

**Figure 3:**
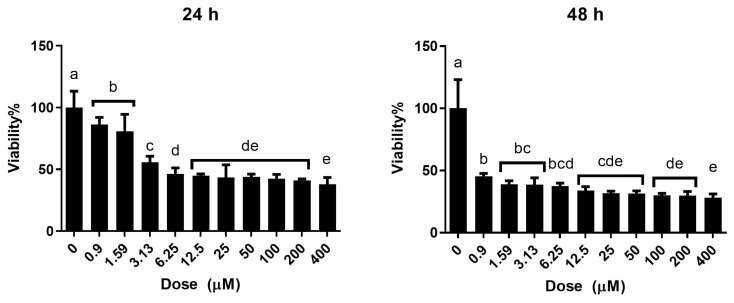
Dose-response curves for % cell viability obtained after 24 h and 48 h of exposure to TiO_2_ NPs in HEL 299/An1 cell line. Different letters indicate a significant difference between means (one-way ANOVA, post hoc Duncan test; ±standard deviation; p < 0.05; n = 8).

TiO_2_ NPs (Sigma 791326) were applied to human embryonic lung cell line HEL 299/An1 for 24 and 48 h at a dose range of 0.9 to 400 μM, as shown in Figure 2. After 24 h of exposure (Figure 3), all doses showed a significant reduction in viability compared to the control group (p < 0.0001), and there was a correlation between decreasing viability and dose (r^2^ = 0.692). The 24-h IC_50_ value was determined to be 25.93 μM. After 48 h of exposure (Figure 3), all doses showed a significant reduction in viability compared to the control group (p < 0.0001), and there was a weak linear relationship between dose and viability (r^2^ = 0.417). The 48-h IC_50_ value was determined to be 0.054 μM.

### 3.2. Determination of titanium quantity by ICP-MS

**Figure 4:**
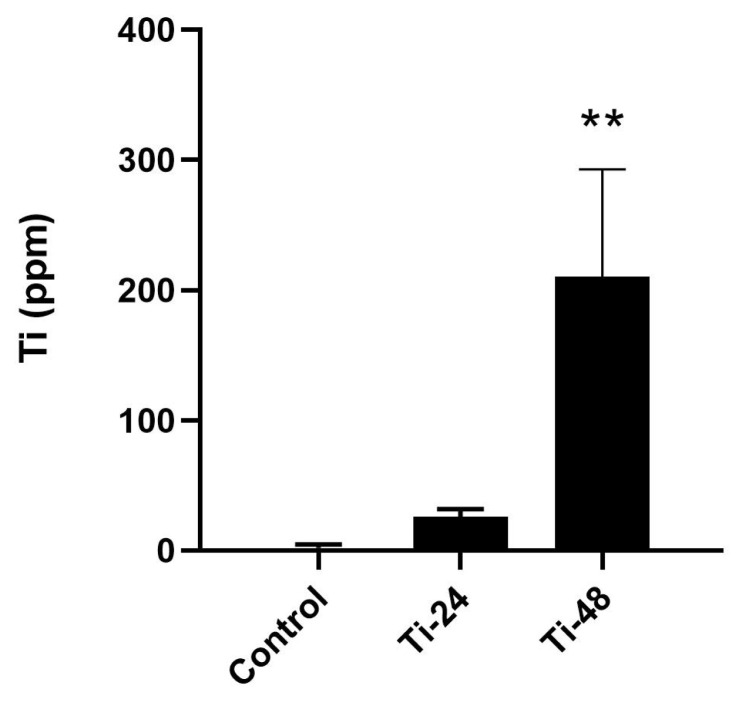
Quantification of TiO_2_ NPs in HEL 299/An1 cells by ICP-MS. One-way ANOVA with post hoc Tukey test (n = 3). **: p < 0.01 compared to control; ±standard deviation.

The ICP-MS data obtained after exposure to the IC_50_ dose for 24 and 48 h are shown in Figure 4. After 48 h of TiO_2_ NP treatment, the amount of titanium in the cells increased compared to the control (p < 0.01). However, the titanium content of the cells was not at a significant level in the group exposed for 24 h.

### 3.3. Determination of 8-OHdG quantity by LC-MS/MS

**Figure 5:**
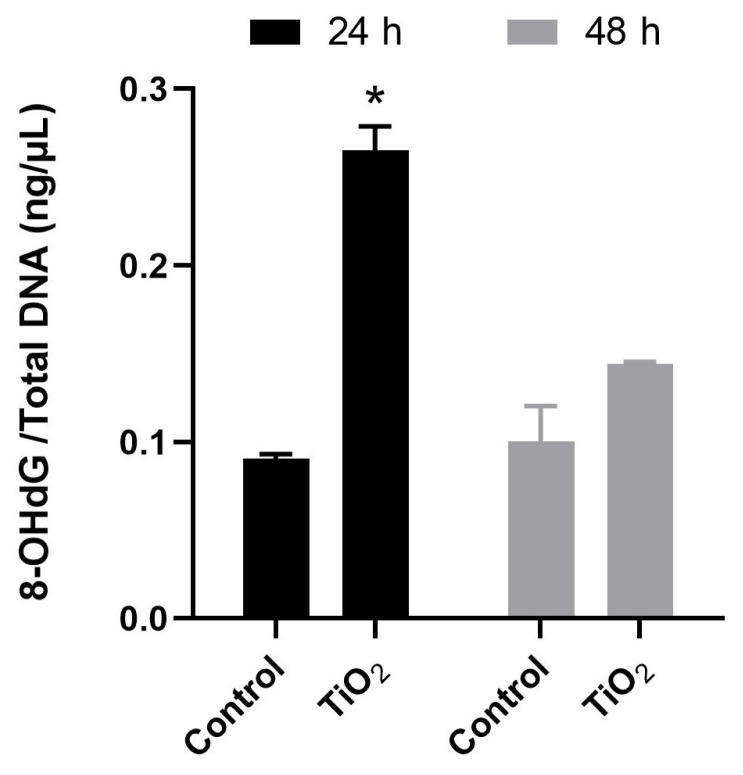
Analysis of 8-OHdG levels by LC-MS/MS after exposure to TiO_2_ NPs for 24 and 48 h. ±Standard deviation (independent variable t-test, *: p < 0.05 compared to the control group for each time point).

The mass fingerprint of 8-OHdG in LC-MS/MS analysis is shown in Figure 2. The amount of 8-OHdG obtained by LC-MS/MS analysis after exposure to the IC_50_ dose for 24 and 48 h is shown in Figure 5. An increase in the amount of 8-OHdG compared to the control group was determined because of exposure to TiO_2_ NPs for 24 h (p < 0.05).

## 4. Discussion

According to the data obtained in our study, brookite TiO_2_ NPs smaller than 100 nm cause cytotoxic effects in human embryonic lung cells by inducing oxidative DNA damage in a time-dependent manner. Exposure to TiO_2_ NPs during pregnancy through various routes may affect placental function and TiO_2_ NPs can reach embryonic lung cells via blood circulation by diffusion or endocytosis [30]. For that reason, we chose to use a human embryonic lung cell line in our study. TiO_2_ NP exposure during pregnancy through the oral route between days 6 and 12 in rats can lead to apoptosis and morphological abnormalities in the offspring’s lungs, which could cause a predisposition to respiratory diseases in the future [31]. In a study of pregnant mice, application of TiO_2_ through intratracheal instillation on days 2.5, 9.5, and 16.5 of pregnancy resulted in decreased expression of matrix metalloproteinase 9 and vascular endothelial growth factor α, which has a significant role in pulmonary vascularization during the fetal period, causing lung development abnormalities in the offspring [25]. Chronic maternal TiO_2_ NPs exposure caused abnormal increases in respiratory rate [32].

In our study, the IC_50_ values for 24 h and 48 h were significantly different, with cells being more sensitive to the nanoparticles at 48 h. There are several possible mechanisms that could explain this difference. One possible mechanism is that nanoparticles can damage cells in a time-dependent manner. At 24 h, the damage may not be severe enough to kill the cells, while at 48 h, the damage may be severe enough to cause cell death.

Another mechanism to consider is the accumulation of titanium. Using ICP-MS, we observed an increased accumulation of titanium in human embryonic lung cells over time. At 24 h, the cells may not have had sufficient time to accumulate a significant amount of TiO_2_ nanoparticles. However, at 48 h, the cells may have accumulated a substantial quantity of TiO_2_ nanoparticles, resulting in cellular damage. This indicates that nanoparticle accumulation within the cells takes time and contributes to cellular damage. The amount of titanium taken into the cells also increased over time, suggesting that the cells may become more sensitive to the nanoparticles over time. This could be due to a number of factors, such as the release of inflammatory cytokines or the activation of stress pathways.

The TiO_2_ NPs taken in by the cells were found to be agglomerated in the cytoplasmic vesicles and sometimes attached to the agglomerate membrane [33]. The amount of titanium taken in by cells is affected by various factors. A previous study found that 11% of TiO_2_ NPs in the anatase crystal phase were located in the cytosol and 4% in the nuclei of human nasal mucosa cells after 24 h of exposure, but it was also stated that DNA damage was not revealed by the comet test and there was no decrease in cell viability [34]. Another study showed that TiO_2_ NPs smaller than 100 nm did not enter the nucleus but remained in the cytoplasm [35]. Titanium salts have low solubility and bioavailability in living organisms, causing them to have a more local effect [36].

Embryos are sensitive to the effects of toxic substances because their defense systems have not yet fully developed. Research has demonstrated that exposure to TiO_2_ NPs leads to an elevation in the amount of oxidants like hydrogen peroxide, superoxide anions, 8-OHdG, and malondialdehyde, while reducing levels of antioxidants such as glutathione peroxidase, glutathione, catalase, and superoxide dismutase. This shift in the balance between antioxidants and oxidants can result in oxidative damage in the tissues of animals [37].

Our study investigated the impact of TiO_2_ NPs on embryonic lung cells by measuring the amount of 8-OHdG. After 24 h of exposure to TiO_2_ NPs, there was a noticeable rise in oxidative DNA damage according to our findings. These findings are consistent with previous research conducted on rats, which showed that exposure to anatase TiO_2_ NPs during the prenatal period resulted in reduced antioxidant enzyme levels while hippocampus lipids and nucleic acids had increased oxidative damage [38]. In other studies, it has been observed that small, spherical TiO_2_ NPs can cause oxidative DNA damage and single-strand breaks, and they can hinder the repair of such damage by inhibiting nucleotide and base excision repair enzymes in the A549 cell line [39]. However, there are also studies stating that exposure to TiO_2_ NPs did not cause DNA damage, which is different from our study [34]. This difference in results may be due to differences in crystal structures, particle sizes, doses, cell types, or the type of DNA damage. Another study reported that anatase TiO_2_ NPs smaller than 100 nm are more sensitive to cytotoxic and genotoxic effects in human lung fibroblast cells (IMR-90) compared to human bronchial epithelial cells (BEAS-2B) and DNA strand breaks were not observed in either cell line, but a high amount of 8-OHdG was detected in the IMR-90 cell line [35]. In a study conducted by Demir et al., DNA strand breaks and micronucleus formation were evaluated using comet and micronucleus tests, respectively, after the exposure of cells to TiO_2_ NPs of 21 and 50 nm in size. These tests were only conducted at the maximum dose of 1000 μg/mL [40]. However, the formamidopyrimidine DNA glycosylase (FPG)-modified comet assay, which detects oxidative purines converted to breaks by the FPG enzyme, did not detect oxidative DNA damage at doses of 10, 100, and 1000 μg/mL. Similarly, at doses of 10 and 100 μg/mL, no micronuclei or DNA chain breaks were detected [40].

We investigated the effects of brookite TiO_2_ NPs on oxidative DNA damage in human embryonic lung cells using LC-MS/MS. We found that the levels of oxidative DNA damage marker 8-OHdG were significantly higher in the 24-h exposure group than in the 48-h exposure group. There are a few possible explanations for the higher levels of oxidative DNA damage in the 24-h exposure group, even though the IC_50_ value and titanium levels were lower in this group. It is possible that nanoparticles cause damage through different mechanisms at different time points. For example, the nanoparticles may be causing cell death at 48 h, but they may be causing oxidative damage at 24 h. The nanoparticles may be more toxic to certain cell types at certain time points. It is also possible that the cells are repairing the damage caused by the nanoparticles over time. For example, the cells may be able to repair the damage caused by the nanoparticles at 24 h, but they may not be able to repair the damage caused by the nanoparticles at 48 h. Oxidative DNA damage is a type of damage that can be caused by nanoparticles. It is caused by the production of free radicals, which are unstable molecules that can damage cells. In this case, the levels of oxidative DNA damage were higher in the 24-h exposure group, suggesting that the nanoparticles were causing more oxidative damage at this time point.

The results of this study suggest that nanoparticles can cause oxidative DNA damage even when they are not causing cell death. This is important to note, as oxidative DNA damage can lead to a number of health problems, including cancer. More research is needed to fully understand the mechanisms by which brookite TiO_2_ NPs damage cells and cause oxidative damage and to develop strategies to prevent this damage. One limitation of our study was the small sample size. Future studies with larger sample sizes are needed to confirm the findings of our study. Furthermore, there is a need for in vivo studies to complement the in vitro findings. Animal models can provide a more comprehensive understanding of the systemic effects of brookite TiO_2_ NPs, including their distribution, accumulation, and long-term implications. In vivo studies can also assess potential biodistribution patterns, metabolisms, and elimination routes, which are crucial for evaluating the overall safety profile and supporting regulatory decisions.

There are health concerns about the environmental impact of nanoparticle waste products. Green nanomaterials are nanoparticles that are synthesized using environmentally friendly methods that do not generate harmful waste products [41]. One example of the studies on this subject is the synthesis of nanosilver particles from olive leaf extract to obtain environmentally friendly and safer metal nanoparticles [41]. One way that green nanomaterials can be used to target cells is by coating them with targeting molecules that bind to specific receptors on the cells [10]. Once the nanomaterials bind to the cells, they can release their payload, such as drugs or genes, directly into the cells. This can be done to treat a variety of diseases, including cancer and genetic disorders. The use of green nanoparticles to target mitochondria is a promising new approach for the delivery of drugs and other therapeutic agents to cancer cells. Mitochondria are the powerhouses of the cell, and they play a critical role in cellular metabolism. By targeting the mitochondria, green nanoparticles can kill cancer cells while sparing healthy cells. This could lead to new and more effective treatments for cancer [42]. There are a few different mechanisms by which green nanoparticles can be targeted to mitochondria. One mechanism is to use ligands that bind to specific receptors on the mitochondrial membrane. For example, the mitochondrial membrane protein translocator protein 18 (TSPO) has been identified as a potential target for mitochondria-targeted nanoparticles [43]. TSPO is overexpressed in cancer cells, making it a potential target for cancer therapy [43]. Another mechanism for targeting mitochondria with green nanoparticles is to use nanoparticles that are made up of materials that accumulate in mitochondria [44]. Once inside the mitochondria, gold nanoparticles can release their payload of drugs or other therapeutic agents [44]. Finally, green nanoparticles can also be targeted to mitochondria by using nanoparticles that are conjugated to specific molecules that are known to accumulate in mitochondria.

In our MTT test for cytotoxicity analysis, we found that cell viability decreased dose-dependently after exposure for 24 and 48 h. We did not induce the TiO_2_ NPs with UV light in order to avoid an increase in cytotoxicity. There may be differences in sensitivity to the cytotoxic effects caused by TiO_2_ NPs between cell lines, but a study of the Hep-2 human cervix carcinoma cell line reported that the cytotoxic effect caused by TiO_2_ NPs was similar to our findings in that it was dependent on dose and time, and the genotoxic effect increased with increasing dose [45].

In conclusion, exposure to TiO_2_ NPs resulted in cytotoxicity in human embryonic lung cells after 24 and 48 h of exposure. There was a significant accumulation of titanium in cells after 48 h. Oxidative DNA damage was observed after 24 h of exposure. The data obtained in this study suggest that exposure to TiO_2_ NPs may lead to negative health effects and bioaccumulation, creating health risks in human embryonic lung cells. It is necessary to take preventive measures to prevent exposure to TiO_2_ NPs, especially for pregnant women who may not be aware of their pregnancy.

## References

[b1-turkjmedsci-53-6-1648] Jana SK, Banerjee P, Das S, Seal S, Chaudhury K (2014). Redox-active nanoceria depolarize mitochondrial membrane of human colon cancer cells. Journal of Nanoparticle Research.

[b2-turkjmedsci-53-6-1648] Warheit DB, Donner EM (2015). Risk assessment strategies for nanoscale and fine-sized titanium dioxide particles: recognizing hazard and exposure issues. Food and Chemical Toxicology.

[b3-turkjmedsci-53-6-1648] Bowdridge EC, Abukabda AB, Engles KJ, McBride CR, Batchelor TP (2019). Maternal engineered nanomaterial inhalation during gestation disrupts vascular kisspeptin reactivity. Toxicological Sciences.

[b4-turkjmedsci-53-6-1648] Shakeel M, Jabeen F, Shabbir S, Asghar MS, Khan MS (2016). Toxicity of nano-titanium dioxide (TiO_2_-NP) through various routes of exposure: a review. Biological Trace Element Research.

[b5-turkjmedsci-53-6-1648] Senzui M, Tamura T, Miura K, Ikarashi Y, Watanabe Y (2010). Study on penetration of titanium dioxide (TiO_2_) nanoparticles into intact and damaged skin in vitro. Journal of Toxicological Sciences.

[b6-turkjmedsci-53-6-1648] Bennat C, Müller-Goymann CC (2000). Skin penetration and stabilization of formulations containing microfine titanium dioxide as physical UV filter. International Journal of Cosmetic Science.

[b7-turkjmedsci-53-6-1648] Allen NS, Mahdjoub N, Vishnyakov V, Kelly PJ, Kriek RJ (2018). The effect of crystalline phase (anatase, brookite and rutile) and size on the photocatalytic activity of calcined polymorphic titanium dioxide (TiO_2_). Polymer Degradation and Stability.

[b8-turkjmedsci-53-6-1648] Ijadpanah-Saravy H, Safari M, Khodadadi-Darban A, Rezaei A (2014). Synthesis of titanium dioxide nanoparticles for photocatalytic degradation of cyanide in wastewater. Analytical Letters.

[b9-turkjmedsci-53-6-1648] Sagadevan S, Imteyaz S, Murugan B, Anita Lett J, Sridewi N (2022). A comprehensive review on green synthesis of titanium dioxide nanoparticles and their diverse biomedical applications. Green Processing and Synthesis.

[b10-turkjmedsci-53-6-1648] Jafari S, Mahyad B, Hashemzadeh H, Janfaza S, Gholikhani T (2020). Biomedical applications of TiO_2_ nanostructures: recent advances. International Journal of Nanomedicine.

[b11-turkjmedsci-53-6-1648] Batra B, Yadav S, Kalra V, Sharma M, Rana JS (2023). An electrochemical biosensor for the determination of folic acid in pregnant women based on DHFR/c-MWCNTs/TiO2NPs modified gold electrode. Sensors International.

[b12-turkjmedsci-53-6-1648] Razali MH, Ismail NA, Yusoff M, Jana S, Jana S (2022). Bio-nanocomposite of carrageenan incorporating titanium dioxide nanoparticles scaffold and hydrogel for tissue engineering applications. Nanoengineering of Biomaterials.

[b13-turkjmedsci-53-6-1648] Kreyling WG, Semmler-Behnke M, Möller W (2006). Health implications of nanoparticles. Journal of Nanoparticle Research.

[b14-turkjmedsci-53-6-1648] Zhu RR, Wang SL, Chao J, Shi DL, Zhang R (2009). Bio-effects of nano-TiO_2_ on DNA and cellular ultrastructure with different polymorph and size. Materials Science and Engineering C.

[b15-turkjmedsci-53-6-1648] Sager TM, Kommineni C, Castranova V (2008). Pulmonary response to intratracheal instillation of ultrafine versus fine titanium dioxide: role of particle surface area. Particle and Fibre Toxicology.

[b16-turkjmedsci-53-6-1648] Liu R, Zhang X, Pu Y, Yin L, Li Y (2010). Small-sized titanium dioxide nanoparticles mediate immune toxicity in rat pulmonary alveolar macrophages in vivo. Journal of Nanoscience and Nanotechnology.

[b17-turkjmedsci-53-6-1648] Gea M, Bonetta S, Iannarelli L, Giovannozzi AM, Maurino V (2019). Shape-engineered titanium dioxide nanoparticles (TiO_2_-NPs): cytotoxicity and genotoxicity in bronchial epithelial cells. Food and Chemical Toxicology.

[b18-turkjmedsci-53-6-1648] Nasr R, Hasanzadeh H, Khaleghian A, Moshtaghian A, Emadi A (2018). Induction of apoptosis and inhibition of invasion in gastric cancer cells by titanium dioxide nanoparticles. Oman Medical Journal.

[b19-turkjmedsci-53-6-1648] Hsiao IL, Huang YJ (2011). Effects of various physicochemical characteristics on the toxicities of ZnO and TiO_2_ nanoparticles toward human lung epithelial cells. Science of the Total Environment.

[b20-turkjmedsci-53-6-1648] Han B, Pei Z, Shi L, Wang Q, Li C (2020). TiO_2_ nanoparticles caused DNA damage in lung and extra-pulmonary organs through ROS-activated FOXO_3a_ signaling pathway after intratracheal administration in rats. International Journal of Nanomedicine.

[b21-turkjmedsci-53-6-1648] Urbaniak SK, Boguszewska K, Szewczuk M, Kazmierczak-Baranska J, Karwowski BT (2020). 8-Oxo-7,8-dihydro-2′-deoxyguanosine (8-oxodG) and 8-hydroxy-2′-deoxyguanosine (8-OHdG) as a potential biomarker for gestational diabetes mellitus (GDM) development. Molecules.

[b22-turkjmedsci-53-6-1648] Shabbir S, Kulyar MF, Bhutta ZA, Boruah P, Asif M (2021). Toxicological consequences of titanium dioxide nanoparticles (TiO_2_NPs) and their jeopardy to human population. BioNanoScience.

[b23-turkjmedsci-53-6-1648] Yamashita K, Yoshioka Y, Higashisaka K, Mimura K, Morishita Y (2011). Silica and titanium dioxide nanoparticles cause pregnancy complications in mice. Nature Nanotechnology.

[b24-turkjmedsci-53-6-1648] Hong F, Zhou Y, Zhao X, Sheng L, Wang L (2017). Maternal exposure to nanosized titanium dioxide suppresses embryonic development in mice. International Journal of Nanomedicine.

[b25-turkjmedsci-53-6-1648] Paul E, Franco-Montoya M, Paineau E, Angeletti B, Vibhushan S (2017). Pulmonary exposure to metallic nanomaterials during pregnancy irreversibly impairs lung development of the offspring. Nanotechnology.

[b26-turkjmedsci-53-6-1648] Wu Y, Chen L, Chen F, Zou H, Wang Z (2020). A key moment for TiO_2_: prenatal exposure to TiO_2_ nanoparticles may inhibit the development of offspring. Ecotoxicology and Environmental Safety.

[b27-turkjmedsci-53-6-1648] Türker NP, Bağcı U, Onsekizoğlu-Bağcı P (2019). Investigation of the anticancer and proliferative effect of broccoli extract on Du145 prostate cancer and MEF healthy fibroblast cell lines. International Journal of Innovative Approaches in Agricultural Research.

[b28-turkjmedsci-53-6-1648] Türker NP, Bağcı U (2019). Effect of quinoa plant on metastasis and ion channels of rat brain cancer glioma cell lines. International Journal of Innovative Approaches in Agricultural Research.

[b29-turkjmedsci-53-6-1648] Crow B, Bishop M, Kovalcik K, Norton D, George J (2008). A simple and cost effective method for the quantification of 8-hydroxy-2⊠deoxyguanosine from urine using liquid chromatography tandem mass spectrometry. Biomedical Chromatography.

[b30-turkjmedsci-53-6-1648] Tedja R, Lim M, Amal R, Marquis C (2012). Effects of serum adsorption on cellular uptake profile and consequent impact of titanium dioxide nanoparticles on human lung cell lines. ACS Nano.

[b31-turkjmedsci-53-6-1648] Elbastawisy YM, Almasry SM (2014). Histomorphological evaluation of maternal and neonatal distal airspaces after maternal intake of nanoparticulate titanium dioxide: an experimental study in Wistar rats. Journal of Molecular Histology.

[b32-turkjmedsci-53-6-1648] Colnot E, Cardoit L, Cabirol MJ, Roudier L, Delville MH (2022). Chronic maternal exposure to titanium dioxide nanoparticles alters breathing in newborn offspring. Particle and Fibre Toxicology.

[b33-turkjmedsci-53-6-1648] Janer G, Del Molino EM, Fernández-Rosas E, Fernández A, Vázquez-Campos S (2014). Cell uptake and oral absorption of titanium dioxide nanoparticles. Toxicology Letters.

[b34-turkjmedsci-53-6-1648] Hackenberg S, Friehs G, Froelich K, Ginzkey C, Koehler C (2010). Intracellular distribution, geno- and cytotoxic effects of nanosized titanium dioxide particles in the anatase crystal phase on human nasal mucosa cells. Toxicology Letters.

[b35-turkjmedsci-53-6-1648] Bhattacharya K, Davoren M, Boertz J, Schins RP, Hoffmann E (2009). Titanium dioxide nanoparticles induce oxidative stress and DNA-adduct formation but not DNA-breakage in human lung cells. Particle and Fibre Toxicology.

[b36-turkjmedsci-53-6-1648] Robertson TA, Sanchez WY, Roberts MS (2010). Are commercially available nanoparticles safe when applied to the skin?. Journal of Biomedical Nanotechnology.

[b37-turkjmedsci-53-6-1648] An H, Ling C, Xu M, Hu M, Wang H (2020). Oxidative damage induced by nano-titanium dioxide in rats and mice: a systematic review and meta-analysis. Biological Trace Element Research.

[b38-turkjmedsci-53-6-1648] Cui Y, Chen X, Zhou Z, Lei Y, Ma M (2014). Prenatal exposure to nanoparticulate titanium dioxide enhances depressive-like behaviors in adult rats. Chemosphere.

[b39-turkjmedsci-53-6-1648] Jugan ML, Barillet S, Simon-Deckers A, Herlin-Boime N, Sauvaigo S (2012). Titanium dioxide nanoparticles exhibit genotoxicity and impair DNA repair activity in A549 cells. Nanotoxicology.

[b40-turkjmedsci-53-6-1648] Demir E, Akça H, Turna F, Aksakal S, Burgucu D (2015). Genotoxic and cell-transforming effects of titanium dioxide nanoparticles. Environmental Research.

[b41-turkjmedsci-53-6-1648] Ramazanli V, Ahmadov I (2022). Synthesis of silver nanoparticles by using extract of olive leaves. Advances in Biology & Earth Sciences.

[b42-turkjmedsci-53-6-1648] Peng H, Yao F, Zhao J, Zhang W, Chen L (2023). Unraveling mitochondria-targeting reactive oxygen species modulation and their implementations in cancer therapy by nanomaterials. Exploration.

[b43-turkjmedsci-53-6-1648] Samuelson LE, Dukes MJ, Hunt CR, Casey JD, Bornhop DJ (2009). TSPO targeted dendrimer imaging agent: synthesis, characterization, and cellular internalization. Bioconjugate Chemistry.

[b44-turkjmedsci-53-6-1648] Smith RA, Porteous CM, Coulter CV, Murphy MP (1999). Selective targeting of an antioxidant to mitochondria. European Journal of Biochemistry.

[b45-turkjmedsci-53-6-1648] Osman IF, Baumgartner A, Cemeli E, Fletcher JN, Anderson D (2010). Genotoxicity and cytotoxicity of zinc oxide and titanium dioxide in HEp-2 cells. Nanomedicine.

